# Supplementary material for: Detection of pneumonia in children through chest radiographs using artificial intelligence in a low-resource setting: A pilot study
Source: PLOS Digit Health. 2025 Sep 24;4(9):e0000713. doi: 10.1371/journal.pdig.0000713 (PMC12459801; doi:10.1371/journal.pdig.0000713)
Supplement: S1 Text — (DOCX) [file pdig.0000713.s001.docx]

***Detection of Pneumonia in Children through Chest Radiographs using Artificial Intelligence in a Low-Resource Setting: A Pilot Study***

**Supplementary Material**

***Data and Code Repository***

The imaging data collected during the study has been de-identified and available open source at <https://doi.org/10.5281/zenodo.14185822>

The code repository is available at <https://github.com/Taofeeq-T/Under5-Pneumonia-AI-detection>

***Software Libraries, Frameworks, and Packages***

The following software libraries and frameworks were employed for model development and training:

TensorFlow (v2.17.0)[^1^](#_ENREF_1): The primary deep learning framework used for building and training the model.

Keras (included within TensorFlow)[^2^](#_ENREF_2): Used for creating the neural network layers and model structure.

Optuna (v3.6.1)[^3^](#_ENREF_3): Used for hyperparameter tuning.

Pandas (v2.1.4)[^4^](#_ENREF_4): Used for data manipulation and storing training history.

**References**

1. TensorFlow-Developers. TensorFlow. v2.17.0 ed: Zenodo; 2024. p. TensorFlow is an end-to-end open source platform for machine learning. It has a comprehensive, flexible ecosystem of tools, libraries, and community resources that lets researchers push the state-of-the-art in ML and developers easily build and deploy ML-powered applications.

2. Chollet F, and others. Keras [Internet]: Github; 2015. Available from: https://github.com/fchollet/keras.

3. Akiba T, Sano S, Yanase T, Ohta T, Koyama M, editors. Optuna: A next-generation hyperparameter optimization framework. Proceedings of the 25th ACM SIGKDD international conference on knowledge discovery & data mining; 2019.

4. The-pandas-development-team. pandas-dev/pandas: Pandas. 2.1.4 ed: Zenodo; 2023.
