# Supplementary material for: Detection of pneumonia in children through chest radiographs using artificial intelligence in a low-resource setting: A pilot study
Source: PLOS Digit Health. 2025 Sep 24;4(9):e0000713. doi: 10.1371/journal.pdig.0000713 (PMC12459801; doi:10.1371/journal.pdig.0000713)
Supplement: Attachment — Submitted filename: response to reviewers- pneumoniaAI.docx [file pdig.0000713.s003.docx]

May 18, 2025.

The Editor-in-Chief,

PLOS Digital Health Journal

**Title of the Manuscript**: Detection of Pneumonia in Children through Chest Radiographs using Artificial Intelligence in a Low-Resource Setting: A Pilot Study

**Manuscript Identifiers**: [PDIG-D-24-00541] - [EMID:ea53c0392adaa14f]

Thank you for the opportunity to revise and resubmit our manuscript. Please find below our point-by-point response to the reviewers’ comments. We have applied required corrections in the proposal, and highlighted them in red.

**#Reviewer 1**

**Comment 1**: The authors should consider expand their dataset. They can collect larger, more diverse local datasets to improve model robustness. Another benefit of having a larger dataset is that they can consider using some of the local data obtained in Nigeria for finetuning, potentially closing the gap of transfer learning. To improve the performance, the authors may also consider testing alternative architectures such as ensembles or vision transformers (VGG-19 is about 10 years old).

**Authors’ Response**: We agree with the reviewer that expanding the dataset is critical to improve model robustness and reduce the performance gap between internal and external test sets. As noted in the **Discussion (lines 252-253) and Conclusion (lines 292-293)**, we already highlighted the limitation of the small external test dataset and its impact on evaluating generalizability. We emphasize the importance of developing robust, locally relevant datasets in Africa. Given the pilot nature of our study and the resource constraints involved in prospective data collection, our initial aim was to demonstrate the feasibility of such an AI model in a low-resource setting.

We also appreciate the recommendation regarding newer architectures. While our model was based on the well-established VGG-19 CNN to balance performance and interpretability, we explicitly recognized this limitation in the **Discussion (lines 250-255)** and proposed as a future direction.

**Change to Text**: N/A

**#Reviewer 2**

**Comment 1**: The title should precisely declare which research question is solved, but the title of the manuscript is general. It is recommended to clearly categorize the studied work and present the research aim in a precise title.

**Authors’ Response**: We appreciate the concern regarding title specificity. However, we respectfully believe that the current title; “**Detection of Pneumonia in Children through Chest Radiographs using Artificial Intelligence in a Low-Resource Setting: A Pilot Study**” already communicates the key components of the research clearly:

- “Detection of Pneumonia in Children” states the clinical objective of the study.
- “through Chest Radiographs using Artificial Intelligence” specifies the modality and methodology used.
- “in a Low-Resource Setting” highlights the context and geographical relevance, which is central to our research question.
- “A Pilot Study” signals that this is an initial feasibility investigation, not a conclusive or large-scale deployment.

The title therefore captures the research problem, the technical approach, the setting, and the study design, aligning with the scientific expectations of clarity and specificity. We hope this explanation clarifies our rationale for retaining the original title.

**Change to Text**: N/A

**Comment 2**: Abstract contains some sentences but some important aspects of abstract are missing. It should contain problems, challenges, method and evaluation clearly. It is recommended to rewrite the abstract.

**Authors’ Response**: The abstract was structured to follow the recommended “Background, Methods, Results, Conclusion” format used by PLOS Digital Health. The diagnostic problem and contextual challenges are presented in the background; the methodology and evaluation metrics (accuracy, precision, recall, AUC) are clearly stated in the methods and results; and the discussion and conclusion sections highlight the implications of performance disparities and the need for localized AI solutions. We have ensured that each section addresses the core elements mentioned.

**Change to Text**: N/A

**Comment 3**: The structure of the manuscript is not clear. The structure of the manuscript or remaining sections of the manuscript should be introduced at the end of the introduction. Please introduce manuscript sections as an outline at the end of the Introduction.

**Authors’ Response**: The current organization adheres to the required format provided by PLOS Digital Health, which specifies the following structure:

Title, Authors, Affiliations, Abstract, Author Summary, Introduction, Results, Discussion, and Materials and Methods.

**Change to Text**: “The remainder of this paper is structured as follows: first, we present the results of the AI model; next, we discuss the findings and their implications; finally, we detail the materials and methods employed in the study.” (Introduction: lines 120-122)

**Comment 4**: What are the advantages and disadvantages of the previous method? Actually, strong literature is a need for a journal article. There is some explanation about previous works. But I could not find previous works disadvantages.

**Authors’ Response**: We have highlighted extensive literature demonstrating the advantages of CNNs in medical imaging, particularly in pneumonia and COVID-19 detection (lines 96–115). We have now expanded the Introduction to include additional literature outlining known limitations of CNNs, including lack of generalizability and challenges with interpretability.

**Change to Text**: “However, CNNs face several limitations. They require large annotated datasets, which are often unavailable in low-resource settings. Their performance can drop when applied to images from different populations or protocols, raising concerns about generalizability.21 CNNs also lack interpretability, functioning as “black boxes”; a drawback in clinical contexts that demand transparency.” (lines 116-120)

**Comment 5**: It seems the proposed approach is mixing several works. The novel contributions are thus not clarified. Please explain where is the novelty of the proposed method.

**Authors’ Response**: The novelty of our study lies in conducting one of the **first prospective validations of a CNN-based AI model for paediatric pneumonia detection using locally collected chest radiographs in Nigeria**. Unlike previous works that rely solely on internal (public) datasets, we focus on highlighting the **domain shift and generalization challenges** of AI models when applied to low-resource settings. This real-world evaluation on Nigerian imaging data and the performance gap analysis against U.S.-trained models provide insights that have not been extensively studied in the literature. These novel aspects are now explicitly emphasized in the Introduction and Discussion sections.

**Change to Text**: N/A

**Comment 6**: As it mentioned in manuscript, resolution is significant in the proposed method. In preprocessing phase, use local and global features in the proposed method as it Considered in recent work of” Toward image super-resolution based on local regression and nonlocal means. Multimed Tools Appl 81, 23473–23492 (2022). https://doi.org/10.1007/s11042-022-12584-x”.” Then compare the results for different image resolution.

**Authors’ Response**: We appreciate this insightful suggestion. While our study acknowledges the importance of image resolution in model performance, we did not implement super-resolution or resolution comparison due to the pilot nature and resource constraints of the study. However, we have now cited the recommended reference (Multimed Tools Appl 2022) and noted in the **Discussion** that future work could explore local and global feature enhancement techniques, such as super-resolution methods, to assess their impact on classification performance in low-resource imaging environments.

**Change to Text**: “Incorporating super-resolution techniques into the preprocessing pipeline may help compensate for resolution disparities between datasets. For example, Bastanfard, Amirkhani 30 proposed a single-image super-resolution method based on local regression and nonlocal means, which outperforms traditional techniques by enhancing fine structural details without requiring complex hardware. Applying such approaches could improve model robustness in settings where imaging quality varies significantly.” (lines 244-249)

**Comment 7**: As it declared in the manuscript, computational complexity is significant. Compare computational complexity of proposed method with state of the art methods.

**Authors’ Response**: Our model is based on the VGG-19 architecture, which, while relatively older, offers lower computational demands compared to deeper or more complex models such as DenseNet or vision transformers. This makes it more suitable for low-resource settings. We have now added a comparison in the Discussion highlighting that although newer models may yield higher accuracy, they often require greater memory, processing power, and training time; limitations that VGG-19 helps mitigate in our setting.

**Change to Text**: “While more recent models such as DenseNet201 or vision transformers may outperform VGG-19 in accuracy, they typically require significantly more parameters, memory, and training time. In contrast, VGG-19 (with approximately 144 million parameters) offers a more computationally feasible solution for environments where advanced hardware is unavailable, making it a practical choice for pilot studies in low-resource settings.” (lines 260-265)

**Comment 8**: What is the order of the proposed algorithm? Discuss on simplicity of algorithm and compare it with others.

**Authors’ Response**: We appreciate the reviewer’s interest in the algorithmic design. The proposed model is based on the VGG-19 architecture with transfer learning and a shallow custom classification head, making the algorithm relatively simple and efficient. The model uses a fixed input size (224×224) and standard convolutional layers with ReLU activations and max pooling, followed by a fully connected dense layer and dropout before binary classification. This structure results in a computational complexity of approximately O(n·k²·m) per convolutional layer (where n = input size, k = kernel size, m = number of filters), with about 144 million parameters overall. This has already been highlighted in the **Model Development** section of the **Methodology** (418-431)

**Change to Text**: N/A

**#Reviewer 3**

**Comment 1**: The quality of the all figures should be improved.

**Authors’ Response**: Figures have been resubmitted in .tif/.eps in line with journal guidelines.

**Change to Text**: N/A

**Comment 2**: The results analysis section is very weak. Statistical results and various tests need to be examined.

**Authors’ Response**: We thank the reviewer for the comment. As this is a pilot study, we have focused on presenting core performance metrics (accuracy, precision, recall, F1-score, and AUC) which are standard for deep learning model evaluation. Additional statistical testing is beyond the intended scope of this feasibility-focused analysis but will be considered in future studies with larger datasets.

**Change to Text**: N/A

**Comment 3**: The current structure of the paper lacks a logical and coherent flow between different sections. It is recommended to rewrite the introduction, methodology, results, and discussion sections separately and in a logically sequential manner.

**Authors’ Response**: The current organization adheres to the required format provided by PLOS Digital Health, which specifies the following structure:

Title, Authors, Affiliations, Abstract, Author Summary, Introduction, Results, Discussion, and Materials and Methods.

**Change to Text**: “The remainder of this paper is structured as follows: first, we present the results of the AI model; next, we discuss the findings and their implications; finally, we detail the materials and methods employed in the study.” (Introduction: lines 120-122)

**Comment 4**: The conclusion section requires a fundamental revision; in particular, it should explicitly address the limitations of the proposed method.

**Authors’ Response**: We appreciate this suggestion. While the study's limitations are discussed in detail earlier (lines 268-273), we have now expanded the Conclusion explicitly acknowledging key limitations, including the use of a single CNN architecture and the small external test set.

**Change to Text**: “This study is limited by the use of a single CNN architecture and a relatively small external test set, which may affect the generalizability of the findings (lines 315-317)

We hope that these iterations comprehensively address the reviewer’s comments.

Sincerely,

Taofeeq O. Togunwa, *MB;BS*

Principal Investigator
