# Supplementary material for: Detection of pneumonia in children through chest radiographs using artificial intelligence in a low-resource setting: A pilot study
Source: PLOS Digit Health. 2025 Sep 24;4(9):e0000713. doi: 10.1371/journal.pdig.0000713 (PMC12459801; doi:10.1371/journal.pdig.0000713)
Supplement: Attachment — Submitted filename: R2 response to reviewers- pneumoniaAI.docx [file pdig.0000713.s004.docx]

August 13, 2025.

The Editor-in-Chief,

PLOS Digital Health Journal

**Title of the Manuscript**: Detection of Pneumonia in Children through Chest Radiographs using Artificial Intelligence in a Low-Resource Setting: A Pilot Study

**Manuscript Identifiers**: [PDIG-D-24-00541R1] - [EMID:22c1281e95b00542]

Thank you for the opportunity to revise and resubmit our manuscript. Please find below our point-by-point response to the reviewers’ comments. We have applied required corrections in the proposal, and highlighted them in red.

**#Reviewer 4**

**Comment 1**: one way to increase the number of data in your study is using Generative models to artificially increase the sample size, you can talk about such models in discussion or limitation section for future works.

https://www.sciencedirect.com/science/article/abs/pii/S1574013720303853

**Authors’ Response**: Thank you for the suggestion. We have addressed the use of generative models to augment the dataset in the Discussion section, highlighting their potential for future work.

**Change to Text**: “Another often overlooked avenue for future work is the use of generative models, such as generative adversarial networks (GANs), to artificially augment training datasets in data-scarce settings. These models can synthesize realistic chest radiographs, potentially improving model generalization across heterogeneous datasets. Previous work has demonstrated that GAN-generated medical images can expand dataset diversity and help mitigate overfitting in small-sample scenarios.44 Incorporating such techniques may be particularly valuable in low-resource settings like Nigeria, where collecting large, high-quality annotated datasets is challenging.” (lines 326 – 332)

**Comment 2**: this study could use explainable techniques (XAI) to present an illustration of which part did the model mis-diagnosed and where did the most of errors occurred. please talk about this solution in discussion section.

https://www.sciencedirect.com/science/article/pii/S1566253523001148

https://link.springer.com/chapter/10.1007/978-3-031-04083-2_2

**Authors’ Response**: Thank you for your suggestion. We have incorporated a discussion on the potential benefits of using explainable artificial intelligence (XAI) techniques, such as Grad-CAM, to visualize misclassifications and error-prone regions. This addition is now included in the Discussion section, highlighting its relevance for future work.

**Change to Text**: “In addition, future work could incorporate explainable artificial intelligence (XAI) techniques to improve model transparency and clinical trust. Methods such as class activation maps (CAM), Grad-CAM, or other saliency-based visualizations can highlight the specific regions of the chest radiograph that influenced the model’s decision.41, 42 This can help identify systematic sources of error by showing whether the model focused on clinically relevant lung regions or was misled by artifacts and irrelevant structures. Prior studies have demonstrated that XAI visualizations in medical imaging not only facilitate error analysis but also enable clinicians to validate AI outputs and detect biases.43 Applying such approaches in future iterations of our model could provide valuable insight into why misclassifications occur and guide targeted improvements in model design.” (lines 316 – 325)

**Comment 3**: training on the ImageNet can also cause this error: since the its a dataset of non-medical images. this is a potential drawback for models using TL and using similar source and target domains may help reduce this error according to:

https://www.mdpi.com/2072-6694/16/11/2138, https://www.sciencedirect.com/science/article/abs/pii/S1566253523002282

please talk more about the effect of discrepancy between source and target domain

**Authors’ Response**: Thank you for your insightful comment. We have expanded upon the impact of domain discrepancy between ImageNet and medical datasets in the Discussion section. Specifically, we address how the non-medical nature of ImageNet may hinder transfer learning in medical imaging tasks and suggest that using source datasets with characteristics more aligned to the target domain could mitigate this issue.

**Change to Text**: “Importantly, the non-medical nature of ImageNet is a known drawback when applying transfer learning (TL) to medical imaging tasks, as models trained on such data may not generalize well to medical-specific image characteristics. Differences in image textures, anatomical features, and diagnostic patterns between the two domains can result in reduced accuracy when the model is tested on medical images. This critical factor contributing to the model's limited performance on external datasets is the discrepancy between the source and target domains. This may be mitigated by using a source and target domain with more similar characteristics; such as fine-tuning models on a medical-specific dataset, or using domain-adaptive training techniques, could reduce the performance gap.33, 34” (lines 256 – 264)

**#Reviewer 5**

**Comment 1**: As a minor, non-mandatory suggestion, the authors may consider: Clarifying in the discussion that statistical comparisons (e.g., confidence intervals, hypothesis tests) were not included and why.

**Authors’ Response:** Thank you for the suggestion. We have clarified in the Discussion section that statistical comparisons, such as confidence intervals and hypothesis tests, were not included due to the pilot nature of the study and the limited sample size of the external test dataset.

**Change to Text:** “Furthermore, statistical comparisons, such as confidence intervals and hypothesis tests, were not included in this study. This was due to the pilot nature of the study and the limited sample size of the external test dataset, which may not provide sufficient power for robust statistical analysis.” (lines 279 – 282)”

We hope that these iterations comprehensively address the reviewer’s comments.

Sincerely,

Taofeeq O. Togunwa, *MB;BS*

Principal Investigator
